# Effects of Combined Acid-alkali and Heat Treatment on the Physiochemical Structure of Moso Bamboo

**DOI:** 10.1038/s41598-020-63907-7

**Published:** 2020-04-21

**Authors:** Jingjing Gao, Lijie Qu, Jing Qian, Zhenyu Wang, Yajing Li, Songlin Yi, Zhengbin He

**Affiliations:** Beijing Key Laboratory of Wood Science and Engineering, College of Material Science and Technology, Beijing Forestry University, No. 35, Qinghua East Road, Haidian District, Beijing, 100083 P.R. China

**Keywords:** Structural materials, Design, synthesis and processing

## Abstract

To improve the performance of bamboo and increase its utilization value, this study aimed at investigating the effects of impregnation pretreatment and thermal treatment on the structural changes of bamboo. The samples were pretreated in sodium hydroxide or zinc chloride solution, and then treated at 160 °C. The pretreated and control samples were characterized by X-ray diffraction (XRD), thermogravimetric analysis (TG), and Fourier transform infrared spectroscopy (FTIR). The results showed that the cellulose crystallinity and intensity of samples pretreated by ZnCl_2_ could be reduced, but the crystal structure remained the same. As for samples pretreated in NaOH, the crystal structure of fiber was destroyed and the crystallinity was increased significantly. High temperature treatment has little effect on the thermal stability of bamboo. However, after treatment with NaOH and ZnCl_2_, the thermal degradation temperature changed obviously and moved to a lower temperature. ZnCl_2_ pretreatment had influence on the chemical structure of bamboo, while NaOH pretreatment had greater influence on the chemical structure of bamboo.

## Introduction

Bamboo, which is characterized by abundant reserves, fast growth, high strength and rigidity, good processability, and strong local availability, is an important sustainable and renewable non-wood forest resource in China^1–3^. At present, bamboo is widely applied in the production of various kinds of bamboo-based composite materials, such as bamboo plywood, laminated bamboo lumber, oriented strand board, and bamboo plastic composite materials, and these products are all widely applied as building materials^4–8^. However, bamboo, as one of the lignocellulose materials composed of cellulose, hemicellulose and lignin, has many shortcomings. For example, bamboo contains high contents of sugars, starch, and proteins, and is thus susceptible to attack by various molds under high humidity conditions^9^. Like wood and other biological materials, bamboo undergoes (shrinkage and swelling) hygroexpansion, and would change its dimensions with the variation of moisture content in its service conditions. As a result, defects such as shrinkage, cracking, and transformation can occur, thus reducing its value^10,11^.

Thermal modification is environmentally friendly and has been widely applied to decrease the hygroscopicity of wood, improve the dimensional stability, durability, weathering ability, antibacterial performance and other characteristics^12–18^. Additionally, the existing research also indicates that heat treatment can decrease the moisture absorption group content and improve the dimensional stability of bamboo^19^. Moreover, after heat treatment at 160 °C, 180 °C, and 200 °C, the thickness expansion and anti-corrosion performance of Cizhu recombined bamboo can be improved, but the strength performance is decreased^20^. However, due to the complex structure of bamboo, the existing bamboo heat treatment is time consuming, and contributes to environmental pollution due to the high content of saccharides, such as sugars and starch, which generate smoke during the heat treatment process^21^.

Additionally, acid/alkali pretreatment is also widely used in the field of bamboo. Alkali pretreatment can effectively delignify cellulose, chemically expand cellulose and enzymatically saccharify bamboo, and cut off the chemical connection between hemicellulose and lignin, removing most of lignin and hemicellulose^22–27^. Acid treatment can promote hydrolysis of bamboo lignocellulose, increase metal absorption and reduce organic adsorption^28,29^. It has been reported by many researchers that acid/alkali pretreatment has certain effects on crystallinity, chemical structure and thermal stability of bamboo fibers^30^. Li *et al*. used 1% NaOH to study bamboo fiber, the results showed that the surface morphology, crystallinity and chemical elements of bamboo fiber had been changed after alkali pretreatment^31^. Lin *et al*. applied NaOH solutions with different concentrations (4%, 6%, and 8%), and concluded that alkali treatment could dissolve impurities, wax, hemicellulose and lignin of bamboo fiber, which could improve crystallinity but not thermal stability^32^. There are also some studies on the combination of acid/alkali treatment and low temperature heat treatment. At two different temperatures of 117 °C and 135 °C, three pretreatment methods of sodium fiber, sulfuric acid and glycerol are used to investigate the chemical composition and structural characteristics of pretreated bamboo fibers changed, and it is concluded that NaOH pretreatment achieved the best enzyme digestibility at higher temperatures^33^. In addition, studies have shown that when the concentration of NaOH is greater than 12%, the cellulose I start to transform to cellulose II, which is more stable^34^. Sugiman *et al*. studied the effects of 4, 8 and 12% NaOH on crystallinity and chemical composition of bamboo fibers, and reached a consistent conclusion^35^.

Although there are many studies on bamboo heat treatment, the problems of high energy consumption in actual heat treatment process were still need to be solved, thus, the combined acid-alkali was applied to modify moso bamboo to decrease its heat treatment decomposition temperature in this study. Additionally, relatively low concentration acid/alkali pretreatment is studied in terms of changing the crystallinity, chemical structure and thermal stability of bamboo fibers, while rare works had focus on the effects of high-concentration alkali pretreatment on bamboo fiber structure and thermal stability, which could change the physiochemical structure of moso bamboo significantly, and improve bamboo characteristics combining with heat treatment. In this paper, the acid and alkali pretreatment has been applied to solve the problems of high energy consumption in actual heat treatment process. 15% sodium hydroxide and ZnCl_2_ were used to pretreat the specimens respectively, and these specimens were further treated with heat treatment at 160 °C. All the samples were characterized by X-ray diffraction (XRD), thermogravimetric analysis (TG), and Fourier transform infrared spectroscopy (FTIR) to analysis the cellulose crystallinity and intensity, thermal decomposed characteristics and chemical structure.

## Results and discussion

### Materials characterization

The holocellulose, cellulose, 1% NaOH extract, moisture content, and ash content of untreated bamboo were measured as shown in Table 1. Before analysis, the specimens were grinded to smaller than 100 mesh and dried at 103 °C for at least 2 h. The ash content was measured by muffle furnace. All analyses were made in duplicate.

**Table 1:** Basic information of untreated bamboo.

Project analysis	Moisture	holocellulose	cellulose	ash	extractives (1% NaOH)
Content	4.94%	73.56%	37.68%	0.65%	33.83%

### Crystal structure analysis by X-ray diffraction

**Figure 1:**
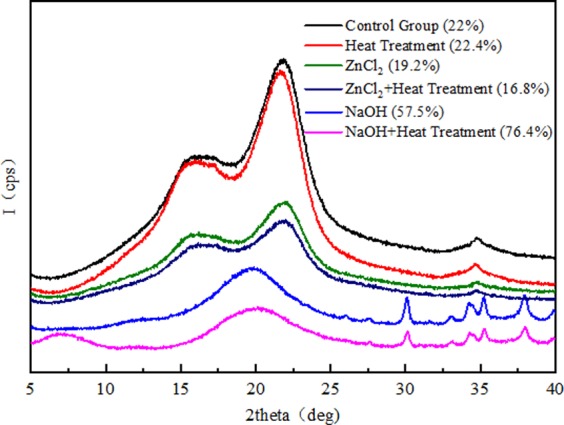
XRD patterns of different samples after pretreatment.

Crystallinity is one of the most important factors in physical and chemical properties. The untreated, pretreated and heat treated samples were analyzed by X-ray diffraction (XRD) to study the crystallization behavior of bamboo powder. In the XRD graph (Fig. 1), the diffraction patterns of bamboo fibers in the non-impregnated group and ZnCl_2_ group exhibit sharp peaks around 2θ = 21.9°, and there are two overlapping weak diffraction peaks at 15° and 16.3°, which are considered to represent typical cellulose I^34^. This indicated that the primary structure of the crystal impregnated with ZnCl_2_ solution was retained, but the intensity varied significantly. However, the diffraction pattern of the bamboo fiber in the NaOH group showed that the crystal structure of the bamboo fiber has changed significantly. This indicated that the crystal structure of bamboo fiber was destroyed, and a new crystal lattice was formed after immersion treatment with NaOH solution^36^. Figure 1 also indicated that the heat pretreatment significantly changed the crystallinity of *Phyllostachys pubescens* under all pretreatment conditions. For the non-impregnated group and ZnCl_2_ group, the effect of heat treatment on the crystal structure and intensity change was not significant. Compared with the other groups, the NaOH group exhibited a significant decreased in intensity after heat treatment. In addition, a new diffraction peak appeared at 2θ = 7°, which indicates that the heat treatment promoted the reformation of the crystal structure of the NaOH group.

**Figure 2:**
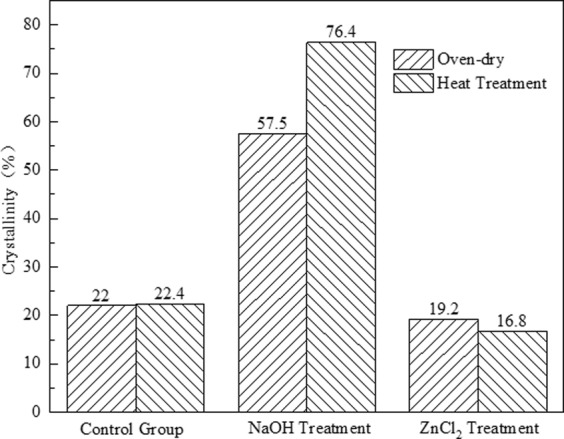
Crystallinity of different samples after pretreatment.

Figure 2 presented the crystallinity index measured by DIFFRAC.EVA software. The samples treated at high temperature showed the most similar crystallinity to the control bamboo (22%), showing 22.4%. This indicated that heat treatment had little effect on the crystallinity of bamboo, which was consistent with previous results. In addition, the crystallinity of the samples treated with NaOH impregnation increased significantly, which may be due to severe hydrolysis on amorphous regions, especially lignin and hemicellulose^37^. In addition, heat treatment promoted the crystallinity of NaOH samples, reflecting the synergistic effect of NaOH and heat treatment. The crystallinity of the sample treated with ZnCl_2_ decreased slightly, which may be due to the degradation of amorphous region of cellulose and even microfibers in the solidification area under the action of acid, resulting in the decrease of the relative crystallinity of bamboo cellulose^1^. Additionally, the results showed that heat treatment significantly reduced the crystallinity of the ZnCl_2_-impregnated samples, reflecting the synergistic effect between heat treatment and ZnCl_2_.

### Thermostability

**Figure 3:**
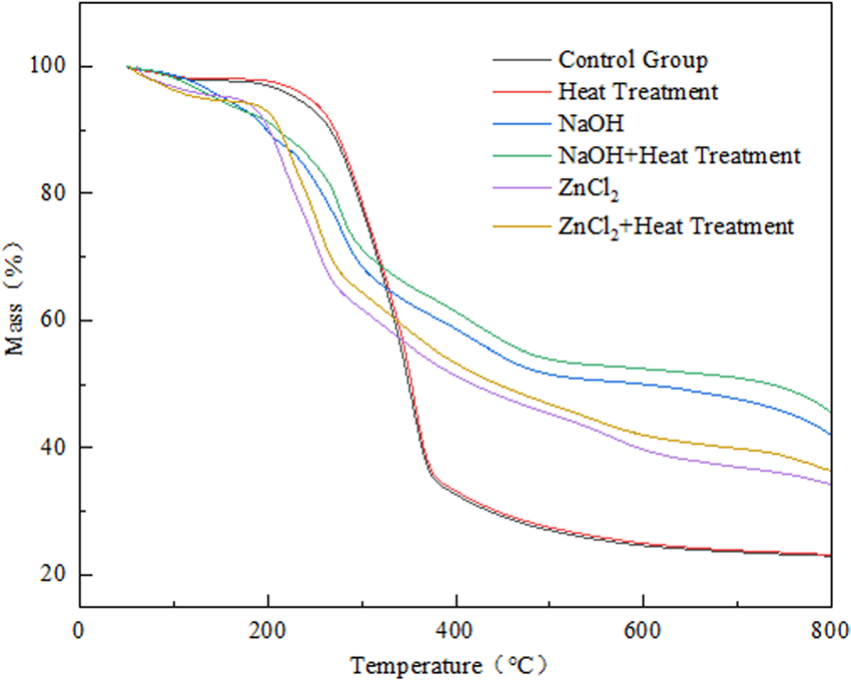
TG curves of different samples at a heating rate of 10 °C/min.

**Figure 4:**
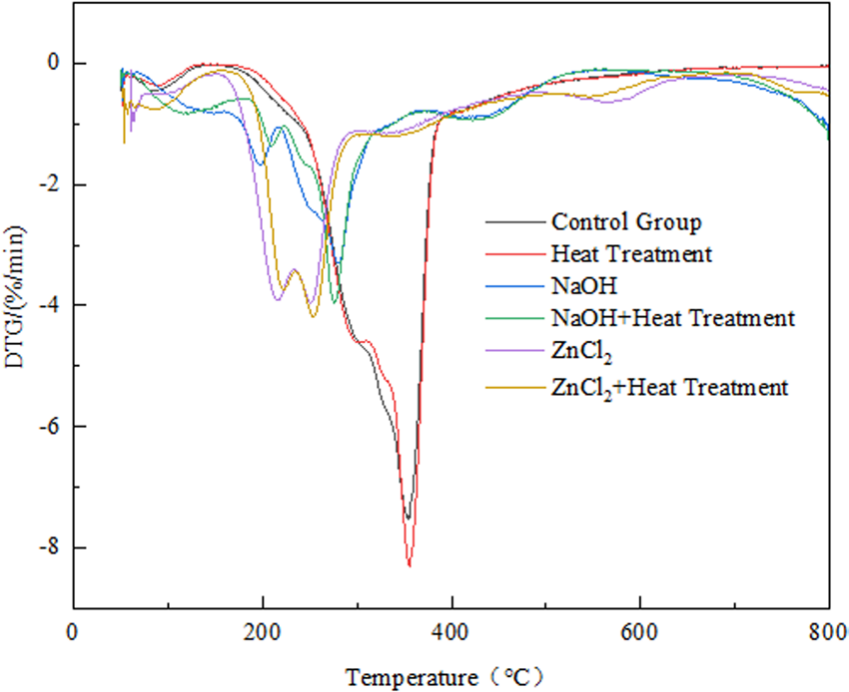
DTG curves of different samples at a heating rate of 10 °C/min.

To evaluate the changes in the thermal properties of *Phyllostachys pubescens* before and after soaking in different solutions and heat treatment, the thermogravimetric (TG) and derivative thermogravimetric (DTG) curves of the bamboo powder treated with NaOH and ZnCl_2_ solutions were presented in Figs. 3 and 4, respectively. Both thermogravimetric curves showed the three weightlessness steps of evaporation of water and light volatiles, decomposition of carbohydrates and pyrolysis of lignin at high temperature^38,39^. It was evident that the thermal stability of bamboo pretreated with different solutions changed significantly, and the heat-treated samples displayed the most similar thermal stability to the control group. Among the thermogravimetric (TG) curves, the degradation process of the heat-treated samples was almost the same as that of the control group, and the amount of decomposition residues was about 23.0%, indicating that high-temperature heat treatment had little effect on the thermal stability of bamboo.

Compared with the control group, the thermal degradation temperature of NaOH and ZnCl_2_ solution varied obviously and were shifted to a lower temperature. For the NaOH group, during the degradation process of the sample, the curve trend changed significantly, the temperature range expanded, and the degradation degree decreased. After pyrolysis, about 42.1% of the mass of bamboo treated by NaOH remained, which was higher than that of the control sample. The increase in residues after treatment with NaOH might be due to the impregnation of the additive into the samples^40^. After heat treatment, the pyrolysis curve of the impregnated samples gradually increased with the increase of temperature, and the final residue content increased to 45.6%, indicating that heat treatment has an impact on the thermal stability of the NaOH-impregnated samples, and there was a synergistic effect between the two. The temperature range in the main decomposition stage of the ZnCl_2_ group was the lowest. The initial curve was similar to that of the control group, and the pyrolysis curve changed significantly with the increase of temperature. After pyrolysis, approximately 34.3% of the mass of the bamboo treated with ZnCl_2_ remained, which was also higher than the control sample. After heat treatment, the pyrolysis curve increased gradually with the increase of temperature, and the residual content increased by 2.1%, reflecting the synergistic effect of heat treatment and ZnCl_2_ solution.

Figure 4 showed the DTG curves of different treated samples and indicates the weight loss rate during thermal degradation. Untreated samples showed more common DTG curves with sharp peaks at 352.8 °C and smaller shoulders at around 300 °C, which may be attributed to the decomposition of hemicellulose and cellulose respectively^41^. Among the DTG curves, the degradation rate of the heat-treated sample was almost the same as that of the control group, except that the maximum weight loss rate increased at 355.3 °C, which indicates that the heat treatment accelerated the decomposition of cellulose and reduces the thermal stability of the sample. Compared with the samples of other heat treatment groups and non-heat treatment groups, the high temperature increased the maximum weight loss rate of the NaOH and ZnCl_2_ groups, indicating that the destruction of bamboo components by heat treatment under the soaking conditions of NaOH and ZnCl_2_ accelerated the decomposition. The maximum weight loss rate of the control group was -7.5%/°C, and the temperature was 352.8 °C. The weight loss rate and weight loss temperature are both higher than those of the NaOH and ZnCl_2_ groups, indicating that the thermal stability changes significantly with the addition of NaOH and ZnCl_2_. The lowest maximum weight loss rate (3.3%/°C) of NaOH samples was found at 280.5 °C, which could be attributed to sodium-induced catalytic devolatilization reaction or hydroxyl ion concentration that affects the thermal stability of bamboo^37^. The ZnCl_2_ group found similar decomposition rates at 214.7 °C and 251.0 °C, respectively, and the weight loss rate was -3.9%/°C. This may be due to the decrease of cellulose content in the sample treated with ZnCl_2_, resulting in a relatively low degradation rate^42^.

### Chemical structure analysis with FTIR spectroscopy

**Figure 5:**
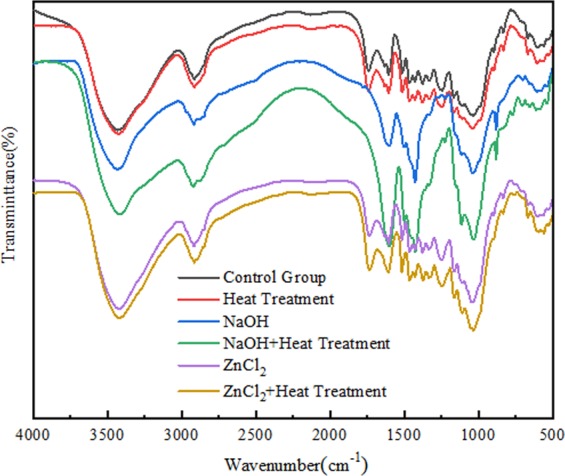
FTIR spectra of different samples after pretreatment.

Fourier transform infrared spectroscopy is a suitable technique, through which the chemical structure changes of samples caused by different treatments can be determined^43^. The FTIR spectra of samples soaked in different solutions were shown in Fig. 5. There are two strong absorption peaks in the 3500–2500 cm^−1^ region, namely the hydroxyl O-H stretching vibration at 3417 cm^−1^ and the C-H stretching vibration on the methyl or methylene group at 2914 cm^−1^, which are abundant in the molecular chains of cellulose, hemicellulose, and lignin^40^. From the figure, it could be seen that the hydroxyl absorption peak intensity of the untreated group was the smallest, and the influence of heat treatment or NaOH impregnation treatment on the change of hydroxyl absorption peak strength was not obvious. However, the influence of joint treatment on the hydroxyl absorption peak strength was significantly increased, indicating that there was a synergistic effect between NaOH and heat treatment. The intensity of the hydroxyl absorption peak in the ZnCl_2_ group was significantly increased compared with the untreated group, but heat treatment had little effect on the ZnCl_2_ group. The C=O group at 1739 cm^−1^ is mainly attributable to hemicellulose xylan, which is a unique component with a relatively high content in hemicellulose, and is easily distinguished from other components^44^. The absorption intensity of this characteristic peak was higher, which indicated that the content of xylan in *Phyllostachys pubescens* was higher. Compared with the untreated group, the peak values of the heat treatment and ZnCl_2_ group did not change significantly, while the peak values of the C and D groups did not change, indicating that the addition of NaOH led to the decomposition of hemicellulose and the significant decrease of sugar. The positions of 1606 cm^−1^ and 1514 cm^−1^ represent C=C groups for which benzene ring skeleton vibration occurs, and these were the main characteristic peaks for studying lignin^44^. The intensity changes of two absorption peaks of untreated and heat-treated wood were not obvious, indicating that the temperature had little effect on lignin. The peaks in groups C and D increased significantly, while those in groups E and F increased slightly, which indicated that the addition of NaOH promoted the formation of benzene rings. In addition, the absorption peaks at 1462 cm^−1^ and 1242 cm^−1^ are mainly from lignin^45,46^. The absorption peaks of the heat treatment and ZnCl_2_ group decreased slightly, indicating that lignin was degraded to some extent. The peak value of the NaOH group was almost zero, which indicated that NaOH had a great influence on lignin degradation. The absorption peaks of C-H at 1375 cm^−1^, C-O-C at 1161 cm^−1^, and C-O-C at 1038 cm^−1^ all come from cellulose and hemicellulose, as does the O-H association absorption band at 1107 cm^−1^ ^44^. It could be seen that the ZnCl_2_ group underwent no obvious change compared with the control group, while the NaOH group had an obvious change in peak value and position, which indicated that the addition of sodium hydroxide had a great influence on the chemical structure of the samples.

## Conclusions

The combination of heat treatment and acid/alkali treatment could effectively improve the physiochemical structure of moso bamboo. Heat treatment after dipping in ZnCl_2_ solution could reduce the crystallinity and cellulose intensity, but could not change its crystal structure. After soaking in NaOH solution and heat treatment, the crystal structure of bamboo fiber was destroyed and the crystallinity was significantly increased. High temperature heat treatment had little effect on the thermal stability of bamboo. However, after treating with NaOH and ZnCl_2_ solution, the thermal degradation temperatures varied distinctly, and were shifted to lower temperatures. The addition of ZnCl_2_ had certain influence on the chemical structure of bamboo, while the addition of NaOH had great influence on the chemical structure of samples.

As shown in this work, acid/alkali pretreatment assisted heat treatment could change the thermal degradation process of bamboo lignocellulose and break its obstinacy, and thus decrease the thermal degradation temperature. With the combination of acid and base pretreatment and heat treatment to reveal the influence on the sample, high concentration (>12%) acid/alkali pretreatment could solve the shortcomings of current bamboo heat treatment, and provide some guidance for bamboo heat treatment for industrial utilization.

## Material and methods

### Sample preparation

Moso bamboo (*Phyllostachys pubescens*) with an age of 3 to 4 years, a moisture content between 5% and 6%, and free from structural defects, such as decay and knots, was obtained from Zhejiang Province. Samples were oven-dried at 103 °C.

### Impregnation pretreatment

Bamboo samples were divided into three groups. Two groups were respectively immersed in NaOH aqueous solution and ZnCl_2_ aqueous solution at 15% w/w, and then treated in a vacuum chamber at 0.002 MPa for 2.0 h. The pressure was then recovered to atmospheric pressure, and vacuuming and the reverse process were performed thrice to impregnate the samples with NaOH and ZnCl_2_, respectively. The control group was not treated.

### Heat treatment

After impregnation with NaOH or ZnCl_2_, all the samples were oven-dried at 103 °C. Each group was divided into two subgroups, one of which was pretreated at 160 °C for 2 h. All the samples were grinded to smaller than 100-mesh before analysis.

### X-ray diffraction analysis

The crystallinity of untreated and pretreated bamboo powder was measured by XRD-6000 X device (Shimadzu), and the supramolecular structure of bamboo after different treatment methods was studied. The filtered monochromatic radiation having a wavelength of 0.154 nm was generated at a voltage of 40 kV and a current of 40 mA. At room temperature, samples were scanned at a scanning speed of 2°/min at an interval of 0.02° in the range of 2θ of 5°–40°. Scattered radiation was detected, and the measured crystallinity index was determined by DIFFRAC.EVA software.

### Thermogravimetric analysis

The degradation characteristics of the raw bamboo powder and bamboo powder pretreated with soaking in different solutions were compared by thermogravimetric analysis. The thermal stability of each sample was measured on a thermogravimetric analyzer (Netzsch STA449F3, Germany), which was operated at a heating rate of 10 °C/min and with a final temperature of 800 °C in an argon environment.

### FTIR analysis

Fourier transform infrared (FTIR) spectroscopy was performed on a standard FTIR spectroscope (VERTEX 70 V, Bruker, Germany). The samples (KBr pellets) for analysis were prepared by mixing 2 mg bamboo powder with 200 mg KBr. Each sample was scanned 32 times and recorded from 4000 to 500 cm^−1^ at a resolution of 4 cm^−1^ in the transmission mode. The background spectrum of pure potassium bromide was subtracted from that of the sample spectrum.
